# The dialogue between what we are living and what we are teaching and learning during Covid-19 pandemic: Reflections of two social work educators from Italy and Spain

**DOI:** 10.1177/1473325020973292

**Published:** 2021-03

**Authors:** Elena Cabiati, Emilio José Gómez-Ciriano

**Affiliations:** Department of Sociology, Catholic University of Milan, Italy, Catholic University of Milan, Milano, Italy; Faculty of Social work, Universidad de Castilla-La Mancha, Cuenca, Spain

**Keywords:** Social work education, resilience, reflective practice, teaching, learning, Covid-19

## Abstract

Italy and Spain have been the most-affected countries in the EU by Covid-19 pandemic. Along with the health, social and economic life of the countries, social work and social work education have been turned upside down. In this essay, the authors reflect on the pandemic’s impact on social work education activities through social work students’ lenses. Accompanying Italian and Spanish students in reflecting on what they were living both, personally and as citizens during Covid-19 and witnessing how, paradoxically, the pandemic offered new opportunities to make important discoveries about key social work issues.

## Introduction

In this short essay we will reflect on the insights shared by students from two southern European universities on how the Covid-19 pandemic^1^ affected them as persons, citizens, students and future practitioners. We will also bring the experiences lived by us as social work educators during the lockdown. How we managed at online theoretical sessions, debates, seminars and evaluation, especially in a discipline- such as social work- on which closeness and contact with students is crucial. This paper, thus, has a lot to do with feelings, hopes and uncertainties. ^2^

*Università Cattolica of Milan* and *Universidad de Castilla-La Mancha* (UCLM) are universities in the Italian and Spanish academic contexts. Both are located in areas severely hit by the Covid-19 pandemic. Both offer social work studies at Graduate level whilst Universitá Cattolica also offers postgraduate courses. Covid-19 pandemic has led to huge changes in the daily lives of both academic communities and as a result a repurposing in the ways of teaching, learning and thinking.

During the months of the lockdown, the authors captured evidence about a fourfold interaction on ’what students were living’, ’what they were being taught and learned’, ’how contents and methodologies were adapted to the situation’ and -last but not least- the way social workers met the needs and welfare structures responded to these need in a context of continuous changes.

**Figure 1.**
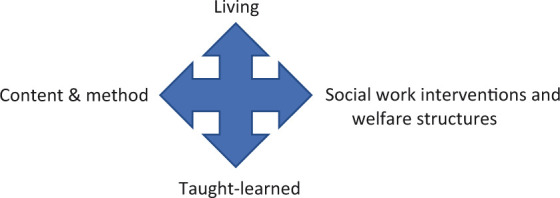
A fourfold interaction during the lockdown.

These four dimensions and their interactions (see Figure 1) fed our teaching and provided us with powerful insights

As Bauman states: ’Fear is the name we give to our uncertaintý to our ignorance of the threat and of what is to be doné (Bauman, 2006: 2). He adds that when `we know where the blow is coming from, we know also what, if anything, we can do to repel it- or at last wève learned, just how limited our ability is to emerge unharmed or what is the kind of loss, or injury, or pain we have to accept’(Bauman, 2006: 1).

During the lockdown, fear and uncertainty were the most common feelings not just among the academic community but in society as a whole. Getting accustomed to keeping in touch virtually, took time and exposed the fragilities in our societies as well as unveiled the dimension of digital divide. This had a decisive impact in the above-mentioned interactions which became intensive, emotional and reflective for social work students and educators.

As social workers-to be, principles of Social Work became particularly relevant during this time, and reflexive practice in social work education became stronger. Accompanying social work students in reflecting on what they were living, both, personally and as citizens during Covid-19, and witnessing how, paradoxically, the pandemic offered them new opportunities to make important discoveries about key social work issues was experimented by us as a precious gift. As suggested by O’Leary and Tsui we must reflect, analyse and learn from this crisis (2020: 274).

## About the situation in Italy and in Spain

*Italy* was the first European country to be affected by COVID-19. The pandemic was mainly located in northern Italy. The eruption of the pandemic caused a never-seen-before disaster in terms of hospitalizations and deaths. The Italian crisis provoked by COVID-19 was considered by analysts as the most serious event in Italian contemporary history after World War II due to its health, social and economic effects.

At the end of February 2020, the University invited all lecturers to suspend all academic activities for one week. This period was extended for another two weeks. These directives have resulted in feelings of confusion and anxiety among students and professors because the seriousness of the situation was not yet clear. During this, teaching was suspended. Placements were also suspended. The expectations of students and professors were that they would be able to retrieve these three weeks before the end of the academic year. However, during the third week of suspension and with the advent of lockdown period (from 10th March to 4th May) the Faculty of Social and Political Sciences got prepared for a period of online lecturing.

The new scenario caught many by surprise. However, despite the first moments of confusion, a strong feeling of commitment emerged. The objective was to overcome the crisis in the better possible conditions for students. The dean of the Faculty of Social Sciences, alongside with professors and administrative staff, worked day and night to offer social work students the best opportunities to follow the educational path Students were also consulted in this phase to express their needs so that they could be accompanied towards a unpredictable scenario.

Every week the faculty updated guidelines to inform students and professors about the situation. The university offered also two virtual platforms through which professors could decide to make online interactive lecturers or video-recording lecturers. some training sessions were also offered.

During this period the resilience of the university was tested, and the community values of the Catholic University were particularly relevant to challenge with the daily consequences of the pandemic.

With regard to Spain, UCLM has traditionally been acknowledged as one of the most technologically advanced universities in Spain. This has to do with its structure as a multicampus university on which the six campuses are distanced more than one hundred kilometers from each other. This previous experience in dealing with IT resources proved to be helpful for remote teaching during the pandemic. However, this did not mean that digital divide not existed. To cope with this, students who had no computers or had difficulties for getting connected were provided by the University with computers, routers and sim cards. Also, many courses were also organized to train academic universities in the new platforms and resources that would be utilized. A deep feeling was in the air particularly among lecturers: How to counterbalance the lack of closeness, with students? how to teach them the importance of face to face contact with users? how to introduce them to the meaning of vulnerability? how to motivate students to continue thinking of their profession as human rights based?. These questions arose and caused a deep concern in us as we felt that, somehow or other the identity of the discipline was being put in question by the pandemic.

The days from March 12th to May 29th were a period of struggle but also of recreation and reinvention on which the contents of the subjects had to be taught on the new light of coronavirus with all it entailed (see Figure 1). This influenced the way we looked at reality, the way reality challenged us and the way we had to introduce this fresh reality to our students in the subjects we taught. (In the case of the Spanish author): `social services programmes and benefits (third course)` and social services and welfare state (first course) ’ both taught in the second semester. In the case of Italy, the subjects taught were: Methodology for social work intervention at case level with students on the second year of the bachelor’s degree, with a module for the methodological elaboration of the practice placement experience that usually occurs between January and May.

## Gaining basic insights into social work practice through a critical dialogue on what we are living and observing

Taking care of people, families and communities in trouble and accompanying them in order to improve/increase their personal wellbeing is supported by the Global Definition of Social Work when it states that ’Social work engages people and structures to address life challenges and enhance wellbeing’ (IFSW and IASSW, 2014), and also by the International Code of Social Work Ethics (IFSW, 2018).

Social work education has as core ambition to prepare students as future professionals in many senses: in learning how to take care of most vulnerable people as future professionals, but also by raising their awareness in detecting oppressive structures and practices that affect human dignity and by promoting social change inspired by principles of social justice, human rights, collective responsibility and respect for diversities.

## Vulnerability

To support others’ wellbeing is often mentioned by social work students as part of the personal motivation in becoming social workers. Sometimes, the same motivations are also rooted in salvific, patronizing attitudes, self-care or professional omniscience.

The pandemic has given people reason to take shock and to recognize their own vulnerability.

In these months, in Italy and in Spain, students and educators have been challenged by unpredictable events that have had an impact on their personal wellbeing and on their ways of teaching and learning. Covid-19 pandemic has thus provided the opportunity to discuss about vulnerability with social work students. This undesirable situation gave to social work teaching activities a particular meaning.

In the online discussion sessions, students were engaged in a *critical dialogue*. We use this term to describe engaging in collaborative and generative consideration about how we want to live and learn together. Our use of the term *dialogue* is influenced by Paulo Freire (1997, 2003), among others. For Freire, critical dialogue is fundamental for developing and acquiring knowledge, as opposed to traditional (and potentially oppressive) practices of indoctrination and passive learning. Knowledge and the learning process are not static, but always in process and in dialogue within the learning environment. The pandemic made this concept more real. Along the different online sessions, the critical dialogue involved students and educators individually and collectively, both as persons and as a community of learners who were actively engaged in a debate on known concepts (social work content), in an unknown context (the pandemic) through new means (the virtual classroom).

The new Covid-19 challenges provided students and educators with the opportunity to critically reflect on what was happening in their life, family and community through social work lenses. The opportunity to reflect not only on ’real problematics’ but also on ’their own problematics’ facilitated a students’ deeper level of analysis and critical reflection.

During Covid-19 pandemic, social work students gained basic insights into social work key concepts that cannot be transmitted only through abstract content.

### Students played as ‘people in need’

The idea of putting oneself into other people’s shoes is over-emphasized in helping professions, sometimes reinforcing false and unrealistic conceptions of empathy (Sevenhuijsen, 2014).

The health emergency that affected Italy and Spain between February and May 2020 helped social work students to raise awareness of their own vulnerabilities when connecting with their personal worries and troubles, experiencing first-hand what means “we are all vulnerable people”. This “all” includes educators, social workers and social work students too.

To play as people in need is an exercise for reflecting on the difficulties of being in trouble and, asking for help. For many students, the lockdown has been an opportunity to play as people in need.

In Italy and in Spain, the Covid-19 pandemic affected social work students: they experienced critical challenges particularly relating to a hospitalized parent or a dead relative, the fragility of family economics, the impossibility to visit the parents living in another Region of the country, the state of stress, anxiety and fear for loved ones.

Recognizing personal weaknesses and speaking about one’s own emotional state is not a simple process for young students, but at the same time, it is a fundamental step in the learning process, necessary to prevent them from suffering from moral blindness and developing resilience as future social workers.

During the online courses, students made explicit their worries and problematics related to Covid-19.

The Covid-19 pandemic encouraged students to spontaneously self-explore in depth naming and sharing needs, feelings and emotions within the virtual classroom, but also in online individual tutorials, in emails sent to the professors or even in their comments written on their assignments on which they publicly or privately expressed their frustrations, hopes and weaknesses:

’*Just write to tell you that in the assignments, in the conclusive part I have shared a personal feeling on how I am living this moment I wanted to do it and I did*’ (student 1)

’*My grandma died two hours ago, and I have no strength to concentrate on my work. This may take out be delayed in my schedule. I beg your understanding*’ (student 2)

’*I am having difficulties when making my assignments on time as we just have one computer and I have to share it with my father who is teleworking* ’ (student 3)

### Students’ learned the importance to take care of themselves first

The need for social workers to be resilient is widely emphasized (Grant and Kinman, 2012) and literature affirmed that building resilience in the future workforce should be a key element of social work education.

However, less is known about the need to develop resilience through self-care as social workers or social workers to be. The topic of developing professionals who are able to deal with the emotional demands of the job is linked to the ability to successfully cope with the personal emotional state. During the Covid-19 pandemic, social work students were exposed to the need to take care of themselves, experiencing that it’s not easy or automatic to be resilient; and that it’s impossible to help others if I’m in personal trouble. During the online courses, they also made explicit that the wish to help others (a neighbor, a citizen in the local community or a person known through the smart-working practice placement experience) was impeded by their own personal worries and sufferings.

Using the words of a student:

’*I’m too worried for myself and for my family that I have no room to help the others*’ (student 4).

This statement, accompanied by feelings of displeasure and blame, gave us the opportunity to reflect with the students about the importance to take care of others’ vulnerability without disregard and neglect of our own wellbeing. The opportunity to discuss about social workers care and wellbeing through the students’ own feelings made it more effective take out the reasons why is important to do it.

## The insights of looking into the social work profession

Social work students have traditionally mirrored social workers as ideal models to be achieved. However, in the Covid-19 context, the mirror depicts distorted images of a profession deeply submerged in an identity crisis (Di Rosa et al., 2019: 121; Duffy, 2015; López Peláez and Gómez-Ciriano, 2019: 145). Students must learn to deconstruct preconceived ideas and discover a different way of being social workers at a context of change on which confusion, uncertainty and doubts are everywhere.

This is not an easy task though, as it requires time, reflection and awareness to identify which tools, concepts and ideas need to be removed, which others can be redefined and repurposed and under which criteria.

So, students will have to go back to the principles on which social work profession are rooted, analyze the way social workers have been performing particularly in times of crisis, and discern new patterns on the light of Covid 19 context.

The inclusion of Human Rights and gender perspective in the content taught online, when analyzing the facts and narratives of what is going on at the streets, hospitals, social services centers, food banks or in the political arena, while participating in discussion groups, when making assignments or when evaluating, helps to raise awareness on this issue and contributes to fill two gaps:

– Firstly, the scarce attention to human rights content in social work curriculum (in Spain is missing and in Italy is sporadic)

– Secondly, the widely shared misconceptions that portray social workers as benefit managers, child catchers or sheriffs, still too extended among the population.

The important role played in Spain and Italy by the respective national colleges/associations of social workers, (Consejo General del Trabajo Social in Spain, Consiglio Nazionale Ordine degli Assistenti sociali in Italy) echoed the voices of professionals strongly committed to the rights of the people in need, denouncing the scarcity of resources and demanding respect for peoplés dignity, these were presented in online sessions through videos and interactive webinars on which students were invited to participate by raising their questions and discussing. For many students who just had vague ideas on what involvement of social workers in politics could mean was very surprising.

Some key questions were raised by students, such as

- If social work is a human rights-based profession and should advocate for the rights of the vulnerable, how is it possible that people are queuing in front of the food banks?
- How is it possible that social workers are working online in social services offices while NGO service delivery is face to face?
- How is the digital divide affecting vulnerable people who are eligible for benefits but do not have either digital skills and or access to Wi-Fi, either or devices in an administration that is not Just digital by default but by necessity?
- Why do politicians not consider social work profession as essential and at the same level as health professions?

## Lessons for the future

Both, in Italy and Spain, retrenchment measures implemented by their respective Governments during the 2018-2014 economic crisis affected structures of welfare and particularly the areas on which social workers were more intensively involved. As a result, social workers had to cope with more demand, less resources and an increasing bureaucratization which provoked stressful and burnout situations in a context of conditionality (Dwyer, 2019) stigmatization of the poor (Cummins, 2018; Gómez Ciriano, 2019) and precariousness of the profession.

Less than six years after the end of the 2018-2019 crisis, it now seems possible that the social and economic effects of Covid-19 may dwarf the effects of the previous crisis, especially when related to the most vulnerable population. A fear spreads amongst our students about whether or not it will be possible for them to work in the stable, dignified and renowned profession they had dreamt of or if quite the contrary they will have to struggle for a decent salary in a growing neoliberal scheme on which individualism, control surveillance and conditionality are the expressions of a businesslike approach of social work (Werzelen et al., 2019: 65). This is something they expressed more frequently as the social effects of the crisis became more and more evident through the weeks of lockdown.

Paradoxically, the lockdown that encapsulated the academic community in their homes during these months demonstrated that Social work is not - and never will be- an encapsulated discipline. The birth of new interactions, the recreation and reinvigoration of old ones. The awareness of how important is to get connected. This connection comprises what students/lecturers are feeling, the responsiveness of the caring initiatives (whether institutional or non-institutional) the pain and hopes of the vulnerable…all this freshness is the composting that makes our plant (social work) grow (see Figure 1) and from which we prepare our lectures.

## Conclusion: Welcoming the unknown and working in undetermined circumstances

Social work is a ‘changing profession’ (Dominelli, 2004) and social work practice is the realm of indeterminism. ‘Indeterminism’ entails unpredictability and low ex-ante control over action (Folgheraiter, 2004: 128).

During Covid-19 pandemic, social work students have seen first-hand what means “to help and to assist in unpredictable and undetermined circumstances”. Observing what was happening to fellow citizens and communities, especially to social workers, practice teachers and volunteers engaged in social welfare institutions and non-profit organizations, students learned what it means to act as a social worker without an accurate pre-vision or accepted standardized process. By reflecting on social work challenges, students observed that social workers were not sure how to proceed in a certain way or were not able to help through a standardized path because during the emergency, social provisions, care assistance and usual professional tools were suspended or distorted.

Through reflection on what social workers were living, students learned that to be proactive in challenging and unpredictable circumstances requires to be able to welcome the unknown and the impossibility of predicting with certainty the flow of the events.

## Notes

1 On 20th of June 2020 the World health organization declared that the confirmed cases are 238.011 (in Italy) and 244.068(in Spain); the deaths are 34.561 (in Italy) and 28.313 (in Spain). For further information: https://covid19.who.int/.

2 Undergraduate social work students of Italy (on the second year of the bachelor program) and on the first and third year in Spain. The groups were around 60 students for each country. The authors teach Methodology of social work, Social services and Welfare State and Social Services programmes and benefits.
